# Gadolinium Pollution—A Future Forward Perspective on Human and Environmental Impact

**DOI:** 10.1002/mrm.70157

**Published:** 2025-10-28

**Authors:** Makayla R. Long, Nir A. Dayan, Meicai Xu, Wei Liao, Assaf A. Gilad, Mark C. DeLano

**Affiliations:** ^1^ Michigan State University College of Human Medicine Grand Rapids Michigan USA; ^2^ Department of Chemical Engineering & Material Science Michigan State University East Lansing Michigan USA; ^3^ Department of Biosystems and Agricultural Engineering Michigan State University East Lansing Michigan USA; ^4^ The Scojen Institute for Synthetic Biology Reichman University Herzliya Israel; ^5^ Department of Radiology Michigan State University East Lansing Michigan USA

**Keywords:** contrast media, environmental, environmental pollutants, gadolinium, magnetic resonance imaging (MRI), remediation, waste disposal

## Abstract

The widespread use of gadolinium‐based contrast agents (GBCAs) in magnetic resonance imaging (MRI) has been instrumental in enhancing diagnostic capabilities. However, the potential environmental and human health impacts of gadolinium pollution have become a growing concern. Despite the stability of GBCAs when bound to organic ligands, gadolinium retention in animals after GBCA administration has been well documented, though the biological impact has been uncertain. GBCAs can degrade and release gadolinium. The degree of dechelation is dependent on the GBCA. The chemical form of the retained gadolinium is likely in the form of a salt. This review presents the current known information about gadolinium pollution as it pertains to both human and environmental health. The long‐term effects of GBCA accumulation in humans most dramatically have been seen in patients with renal insufficiency through the association with nephrogenic systemic fibrosis (NSF), an iatrogenic disease that was not recognized until after more than 15 years of GBCA utilization. Neural deposition in people after repeated exposures has yet to have proven association with neurotoxicity, but it is widely agreed that exposure should be limited to clinical necessity. Environmentally there is concern for gadolinium runoff, contamination of water systems, bioaccumulation, and compounding ecological harm. We propose strategies for mitigating gadolinium pollution, including reducing the use of contrast agents, improving waste disposal/recovery techniques, and advancing research on microbial remediation methods. Addressing gadolinium pollution will be a collaborative, interdisciplinary effort that begins with improving awareness of the problem, which is what this review intends to accomplish here.

## 1 Introduction

Healthcare technology continues to evolve with significant improvements in diagnoses and treatments largely driven by decades of advancements in medical imaging. Since 1988, gadolinium‐based contrast agents (GBCAs) have been used in more than 300 million procedures worldwide to improve image contrast in Magnetic Resonance Imaging (MRIs) [1]. The composition of GBCAs has been improved to be more stable, but many have raised concerns that dissociated gadolinium may have potentially extreme adverse effects on humans, animals, and our environment. The manufacturing process, administration of GBCA, and disposal/excretion of gadolinium create many points of vulnerability that result in potentially toxic pollution. For example, GBCAs are primarily excreted through urine and enter the sewage system, like municipal wastewater treatment plants (WWTPs). This anthropogenic gadolinium is not monitored at WWTPs as a standard practice. Due to the high solubility and stability of GBCAs, they are not effectively removed by conventional treatment processes, leading to their accumulation in surface water and even drinking water supplies [2, 3]. As the utility and demand for gadolinium in MRI applications continue to grow, we encourage greater collective effort among scientists, physicians, and researchers to understand the full scope of gadolinium's effect on human health and the environment. This review seeks to raise awareness of gadolinium pollution and advocate for meaningful interventions, aiming to catalyze change through interdisciplinary research.

## 2 Background

### 2.1 Chemistry and History

Gadolinium (Gd) is one of 15 lanthanide group elements. These elements are called rare earth metals or rare earth elements (REEs). Despite being named “rare,” they are naturally occurring, abundant, and are successfully mined. The lanthanides have a unique electron configuration which makes them highly reactive, including their ability to react with most non‐metal elements. For GBCAs specifically, all agents contain Gd^+3^ which has the most unpaired electrons of any stable ion. This allows enhancement of proton relaxation and creates a high magnetic moment which is what makes it such a favorable contrast agent [4, 5], a measurable characteristic of GBCAs termed relaxivity.

GBCAs are chelated with organic ligands with either linear or macrocyclic structures, with the latter conferring far greater stability. The American College of Radiology (ACR) has classified GBCAs into two groups. First generation, simple linear Group I agents (gadodiamide, gadopentetate, and gadoversetamide) were associated with nephrogenic systemic fibrosis (NSF) and are no longer on the market. Group II agents include the macrocyclic agents and the substituted linear GBCAs gadobenate and gadoxetate. Group II agents have been associated with few if any cases of NSF [6, 7, 8]. The substituent on the gadobenate and gadoxetate molecules results in greater stability than Group I linear agents due to greater steric hindrance to dechelation and a dual route of elimination (renal and biliary excretion). A compilation of the chemical structures of FDA‐approved GBCAs can be found in the review by Le Fur and Caravan [9], which thoroughly discusses the relevance of GBCAs from a bioinorganic chemist perspective. A table summarizing the main properties of clinically approved GBCAs can also be found in the reviews by Scarciglia et al. [10] and Ebrahimi and Barbieri [11]. The properties and dosing recommendations for GBCAs are listed in the review by Lohrke et al. [1] review. It is important to note that when gadolinium is bound to an organic ligand, it is non‐toxic and inert in vivo which has made GBCAs successful, long‐standing MRI contrast agents. However, in relevance to environmental pollution, all GBCAs can degrade, with dissociation of the Gd^+3^ from the chelate by UV radiation [2]. As will be discussed later in this review, this is concerning as GBCAs are primarily eliminated via urine, enter wastewater, are treated via UV radiation, and thus release the toxic component of the GBCA, further compounding the gadolinium pollution problem [12]. Importantly, the biliary elimination of gadoxetate and gadobenate in stools compounds the problem of GBCAs entering wastewater [13, 14]. More history on the development and chemistry of GBCAs can be found at the following sources [1, 9, 10, 15, 16].

## 3 Current Gadolinium Problem

### 3.1 Manufacturing, Supply, Demand

The process from initial mining to injecting a patient with a gadolinium‐based contrast agent (GBCA) is extensive. Gadolinium is first extracted from the earth where it coexists with base metals and other minerals. The raw material undergoes processing to isolate gadolinium, followed by multiple acid wash cycles for purification. This initial stage of gadolinium consumerism is also the first major source of environmental pollution—acid washes create high volumes of gadolinium‐containing waste material that can leach into the ground and drinking water along with other heavy metals and acidic residuals. Improper disposal of these mining effluents has been linked to soil acidification and contamination of groundwater near rare earth element mining sites [17]. Concern for the environment, ecosystem, miners, and human inhabitants of these polluted areas is warranted, although current data on the safety of mining processes and worker health impact is scare to non‐existent. The issue of trustworthy and reliable data from these mining districts is an obstacle to discovery as the top 3 producers of mined and purified gadolinium are China, Russia, and Malaysia—in order respectively [18]. China alone accounts for 98% of the global supply of all rare earth elements [19]. A large‐scale study was performed in 2012 to analyze the statistics of rare earth metal supply, demand, availability, and projected growth. The authors argue that the supply of rare earth elements such as gadolinium is threatened by politically polarized supply conditions, unsustainable mining practices, and rapidly increasing growth and demand for the purified product [18].

Purified gadolinium is predominantly used to make MRI contrast agents, but it is also heavily relied on in the commercial industry to manufacture magnets, electronic devices, computers, and wind turbines. It is also used in nuclear reactor control rods and in the glass industry for polishing lenses [18, 20]. Currently, no comprehensive data exist on the global distribution of gadolinium usage across different sectors, making it difficult to quantify how much is consumed by healthcare versus industrial applications. Nevertheless, the sustainability of gadolinium supply is of strategic importance across these sectors, most notably in healthcare where the demand for contrast‐enhanced MRIs continues to grow. An estimated 30 million MR scans are performed per year worldwide, with approximately 40% utilizing contrast agents [21]. The number of MRI scans performed each year is anticipated to increase due to an aging global population, rising prevalence of chronic conditions, patient‐generated demand, and physicians' defensive practice of overutilization of imaging. Proliferation of MR devices within healthcare systems and easier access have also significantly contributed to the increase in MRI procedure volume. A large study following just over 377 000 participants for 10 years (1997–2006), tracked the number of healthcare imaging modalities performed on them (x‐ray, Mammography, US, CT, and MRI). The study found that imaging increased for every modality over the time period but most notably for MRIs [22]. Additionally, they found the total number of imaging tests increased markedly with age [22], which is concerning and notable in light of GBCA pollution as the global population is rapidly aging. As a follow‐up to the previous study, a more recent exploration of imaging modality rates spanning 2000–2016 was performed and found that CT and MRI use continued to increase, but at a slower pace in more recent years [23]. Although the number of MRI scans does not directly equate to GBCA doses, the overall trend toward increased use of advanced imaging modalities underscores the rising demand for GBCAs. This reinforces the urgency of addressing gadolinium sustainability and its environmental consequences on a national and global front. Further data and trending of USA and 19 other countries between 1995 and 2017 can be found in the review Ebrahimi and Barbieri [11]. To this end, there are countervailing trends to reduce gadolinium exposure which we describe further below.

### 3.2 Gadolinium in the Body

Unchelated gadolinium is regarded as highly toxic in animals due to interference with calcium‐ion channel dependent processes. MRI contrast agents are chelated compounds and are largely considered safe for use in humans. However, it is well documented that gadolinium accumulates in the skin, muscle, bone, and brain [24, 25, 26, 27, 28], and gadolinium retention has also been well documented in several autopsy studies [24, 29]. The biological impact of this deposition is not clear, and the chemical form of the deposited gadolinium is not known and may either remain chelated, be deposited as a salt, or potentially be bound to other macromolecules. Both the molecular environment and the gadolinium‐chelate complex impact stability. Mobilization of gadolinium from the chelate may impart its toxicity. Repeated administration of all GBCAs has been shown to result in tissue deposition to varying degrees.

In a previous mouse study evaluating the effects of GBCA on the central nervous system, GBCAs induced neuronal cell death in the hippocampus which worsened when inflammation was present. In these mice at the peak of experimental neuroinflammation, neurotoxicity was demonstrated with high‐dose administration of a now discontinued linear GBCA (gadopentetate) at 25 times the normal dose using 8 such doses over 10 days [30]. Recognition of high signal intensity in the dentate nucleus and globus pallidus of the brain related to gadolinium retention renewed attention to the issue of incomplete GBCA clearance and raised the question of potential neurotoxicity [29, 31]. However, Vymazal and colleagues [32] showed that no neurological effects were seen in their case series in which some patients received almost a liter of GBCAs over 14 years. It has also been shown that GBCAs inadvertently cause nephrogenic systemic fibrosis (NSF) in patients with preexisting impaired renal function [33, 34]. The advent of NSF changed radiologic practice and prompted the universal adoption of renal function screening prior to administration of contrast for MR exams. This dramatically reduced the incidence of new cases of NSF by restricting contrast from patients with impaired renal function and glomerular filtration rate (GFR) < 30 mL/min. With the adoption of macrocyclic agents, the incidence of NSF has essentially been eliminated, and as a result, screening for renal function is arguably unnecessary in most patients [35]. While the risk of NSF for patients with severe kidney disease has decreased to a point where withholding these agents may cause more harm than good, according to clinical guidelines, other research poses further concerns about long‐term implications such as competition of gadolinium with voltage‐gated Ca^+2^ channels and Ca^+2^ dependent enzymes [36].

The potential health impact of GBCAs is compounded when you consider the prolonged retention of gadolinium in the body. There is conflicting research and data about the elimination rate of GBCAs from the body, with much of the variability due to the specific GBCA, its associated chelate, and the tissue sample. Studies have shown that post‐contrast MRI, a healthy, normally functioning kidney can excrete 100% of the GBCA dose in a few days [37], urinary elimination of GBCA more than 1 month after injection [38], and another older study showed that > 95% of GBCA is eliminated in 72 h [39]. The large discrepancies on GBCA elimination from the body are concerning yet motivating to continue investigating the factors which affect elimination rate, and deciphering what the implications of delayed elimination mean to our health.

The ACR Committee on Drugs and Contrast Media established nomenclature for Symptoms Associated with Gadolinium Exposure, or SAGE to assist in the description of symptoms that are unrelated to acute (hypersensitivity or physiologic reactions) and late‐onset (NSF) adverse effects. SAGE is divided into early‐onset and late‐onset groups and is intended to be used when a causal relationship between GBCA exposure and symptoms is unknown [40]. So‐called gadolinium deposition disease (GDD) reported as a form of gadolinium toxicity remains controversial. Those supporting this entity state that GDD has achieved generalizability, which is a critical requirement for disease recognition. Semelka and Ramalho report that GDD is a distinct diagnostic entity with a constellation of clinical symptoms that has been successfully treated by avoidance of future gadolinium and by chelation with DTPA [41, 42, 43]. Ongoing debate supports further investigation to better understand the pathologic basis of GDD and definitive disease recognition [42, 43, 44, 45, 46]. The increasing restrictions in Europe present another perspective on human and environmental health, and there is considerable ongoing research being conducted around the globe regarding the human and environmental impacts of gadolinium pollution [47].

### 3.3 Human Exposure Limiting Strategies

In an ideal world, diagnoses could be obtained without the use of any gadolinium. To date, many strategies have been adopted to reduce exposure. Among these are the judicious use of contrast only when needed and avoidance of waste as discussed in further detail below. Use of higher relaxivity agents has been shown to allow for half‐dose contrast with preserved diagnostic efficacy [48]. For example, gadopiclenol has the highest relaxivity of all approved agents currently on the market in the US and Europe and is approved at half of the dose of other GBCAs, meaning that half of the amount of gadolinium enters the human body and, subsequently, the environment. Further reductions are likely with the use of artificial intelligence and deep learning to amplify the effects of these contrast agents. Improved agents and dose reduction are areas of ongoing research [49, 50, 51]. The development of non‐contrast MRI mechanisms to generate important contrast differences in tissues such as chemical exchange saturation transfer (CEST) continues to mature. Diamagnetic CEST is becoming increasingly useful for metabolic imaging of tumors. Capacity to monitor glucose metabolism provides insights on tumor aggressiveness and response to therapy. Additionally, other metabolites and pH imaging show potential in the assessment of tumor microenvironments [52, 53, 54]. Decreased medical need and decreased use can dramatically reduce our next topic, the environmental impact.

### 3.4 Gadolinium in the Environment

A European study revealed that despite linear GBCAs comprising less than 10% of contrast enhanced MRI exams in 2017 following regulatory restrictions due to their association with NSF and decomposition concerns [7], elevated levels of gadolinium were still detected in shellfish several years later [12]. This finding underscores the persistence of Gd in aquatic environments, even long after its primary source has been substantially reduced. It is known that GBCAs can be accumulated through the food chain which further emphasizes the importance of healthy aquatic life. A major 2024 study investigated marine gadolinium contamination and its ecological implications. They identified GBCAs used in MRI procedures as the primary source of anthropogenic gadolinium, and the marine gadolinium pollution is primarily caused by GBCA runoff. Over 70% of medically developed regions showed gadolinium anomalies in surface water [55]. In a separate investigation, researchers analyzed tap water in Berlin, Germany, comparing the city's eastern and western districts. While gadolinium concentrations remained stable in the eastern districts between 2009 and 2012, the western districts experienced significant increases, with some areas reporting gadolinium levels up to 11.5 times higher in 2012 than in 2009 [56]. These findings demonstrate the distribution of GBCA‐derived gadolinium in both natural and drinking water systems. This evidence is critical to understanding the environmental distribution of gadolinium and underscores the compelling need for further research, public awareness, and immediate, coordinated proactive intervention.

### 3.5 Waste Management

In addition to reducing overall GBCA usage, a critical yet often overlooked strategy for mitigating gadolinium pollution involves addressing the waste generated during GBCA administration. Once GBCAs are compounded, they are stored in vials for use in MRI procedures. The administration process involves various single‐use items such as needles, syringes, swabs, and tubing which are discarded after use for GBCA administration. These findings raise serious concerns as the long‐term impacts of gadolinium‐laden waste entering regular disposal streams remain poorly understood. The potential interactions of this waste with soil, water, and human health warrant comprehensive investigation and proactive waste management strategies. This raises a critical question: how should we manage GBCA‐contaminated materials? A pivotal 2021 study explored this issue by examining GBCA dosages relative to patient weight and analyzing the disposal of associated materials. The study found that solid waste material such as syringes containing unused GBCA, is typically incinerated and may subsequently be incorporated into products like cement. Alarmingly, approximately 15% of prescribed GBCAs went unused [57]. However, multidose vials and syringeless injectors are available from some contrast vendors which can reduce waste.

### 3.6 Microbial Clean‐Up Alternatives

As early as the 1970s, Sherry and Cottam showed that rare earth elements (REEs), and specifically gadolinium, can displace calcium bound to proteins [58]. Other proteins from a variety of organisms can bind gadolinium directly or with the assistance of co‐factors [59]. A notable example is Lanmodulin, a protein isolated from bacteria that exhibits highly selective binding to lanthanides with picomolar affinity [60]. This discovery led to the development of Lanmodulin‐based biosensors, capable of quantifying REEs [61] or detecting their presence through fluorescence‐based assays [62, 63]. Furthermore, Lanmodulin's lanthanide binding motifs have been leveraged to engineer gadolinium‐binding proteins, which serve as MRI contrast agents [64].

An intriguing approach to clean up environmental gadolinium is inspired by the discovery of lanthanide‐using bacteria over the past decade. Many species use a suite of specialized molecules to bind, transport, and employ these lanthanide elements to activate or potentiate their metabolism. These species of bacteria could potentially be used to discover “hot spots” of lanthanide sources, or used in environmental clean‐up [65]. This presents the opportunity for using naturally occurring lanthanide binding motifs (peptides or proteins incorporating the motifs) or alternatively inventing novel peptides to sequester lanthanides efficiently, even when these are present at very low levels. This attribute can be used to our advantage to make it more commercially viable to extract lanthanides from mine run‐off, medical waste, and electronic waste. Invention of peptides and harnessing biological organisms' natural ability to acquire gadolinium and other REEs have been proposed in use of solving both problems—recycling to support growing demand for REEs, and for environmental toxic cleanup [66].

In 2022, Good and colleagues [67] were the first to demonstrate that *Methylorubrum extorquens* AM1 genetic variant could accumulate and sequester gadolinium at significantly high levels. The bacteria and their genetic variant could acquire gadolinium so significantly that it was able to produce MRI contrast in whole cells. The bacteria were also able to sequester gadolinium from DTPA (diethylenetriamine pentaacetate) which demonstrates that it could be used for purposes of gadolinium recycling and environmental remediation [67].

The review of previous studies investigating microbial clean‐up strategies for lanthanides (Table 1) shows that while significant progress has been made in recycling various REEs using existing technologies, the targeted recovery of gadolinium remains largely unexplored. This represents a critical knowledge gap and a unique opportunity to advance gadolinium recovery at the levels of mining, medical waste management, and environmental remediation.

**TABLE 1:** Microbial clean‐up alternatives for lanthanides.

Bacteria used	Mechanism of action	Lanthanides used	Reference
*Methylorubrum extorquens* AM1	Intracellular Ln sequestration in polyphosphate granules.	Nd	Good et al. [68]
*Methylorubrum extorquens* AM1‐evo‐HLn	This mutant boosts Gd accumulation by altering transcription related to heavy metal stress, methanol oxidation, and metabolism. Upregulated genes involved in Ln acquisition, lanthanophore production, PQQ biosynthesis, and phosphate transport.	Gd and light Ln	Good et al. [67]
*Shewanella oneidensis* MR‐1	Biosorption.	La, Eu, Yb	Medin et al. [69]
*Gluconobacter oxydans* B58	Engineered *G. oxydans* bioleaches lanthanides by secreting organic acids that dissolve minerals, then capturing the released ions via cell‐surface ligands and enhanced metal uptake systems.	Ce, La, Nd, Pr	Schmitz et al. [70]
*Caulobacter crescentus*	High‐density cell surface display of lanthanide binding tags on its S‐layer.	Eu, Yb, Dy, Tb, Y, Nd, Ce, La	Park et al. [71]
*Escherichia coli*	Surface expression of the lanthanide binding protein lanmodulin on *E. coli* .	Y, La, Gd, Tb	Gut et al. [72]
*Methylacidiphilum fumariolicum* SolV	Methanol dehydrogenase enzyme system.	La, Ce, Pr, Nd	Singer et al. [73]
Cyanobacteria, most efficient *Nostoc* sp. *20.02*	Biosorption.	Ce, Nd, Tb, La	Paper et al. [74]

*Note*: Microbial strategies for lanthanide recovery, including biosorption, organic acid bioleaching, and engineered protein‐based binding across light and heavy lanthanides.

Building on laboratory‐scale proof‐of‐concept studies, current research is increasingly focused on translating microbial systems into scalable, real‐world gadolinium recovery applications. For example, bioreactor‐based systems incorporating immobilized or biofilm‐forming lanthanide‐binding bacteria are being investigated for continuous treatment of gadolinium‐contaminated effluents [66]. The integration of synthetic biology tools allows for the optimization of binding motifs and regulatory elements to enhance uptake efficiency and selectivity. Despite this promise, several challenges remain, including the scalability of microbial platforms, biosafety considerations, and economic viability compared to traditional physicochemical approaches. Nonetheless, microbial biosorption stands out as a compelling complementary strategy to enhance existing treatment frameworks and support the circular recovery of REEs from both medical and industrial waste streams.

### 3.7 Advanced Water Treatment Strategies

The increasing prevalence of GBCAs in global water systems has prompted significant interest in developing advanced water treatment technologies to mitigate their environmental impact. Conventional wastewater treatment plants (WWTPs) are not equipped to effectively remove these highly stable chelates that bypass typical treatment processes, resulting in their persistence in surface water and in drinking water supplies [37]. To address this challenge, a range of physiochemical treatment methods has been explored, including nanofiltration, reverse osmosis (RO), adsorption, and ion‐exchange processes [2]. Among these, RO demonstrates the highest removal efficiency with studies reporting removal efficiencies as high as 99.85% for anthropogenic gadolinium [75]. However, the high‐pressure operation and energy demands of RO systems present barriers to large‐scale adoption, especially in municipal settings.

Adsorption‐based techniques have also shown promise, particularly for removing free Gd^+3^ ions and degradation byproducts of GBCAs. Materials such as activated carbon, biochar, and engineered polymer resins are being investigated for their capacity to capture these compounds through surface binding and ion exchange mechanisms [76]. These methods are generally more scalable and cost‐effective than membrane processes, though their efficiency can vary based on the specific chemical form of gadolinium and the characteristics of the adsorbent.

**FIGURE 1:**
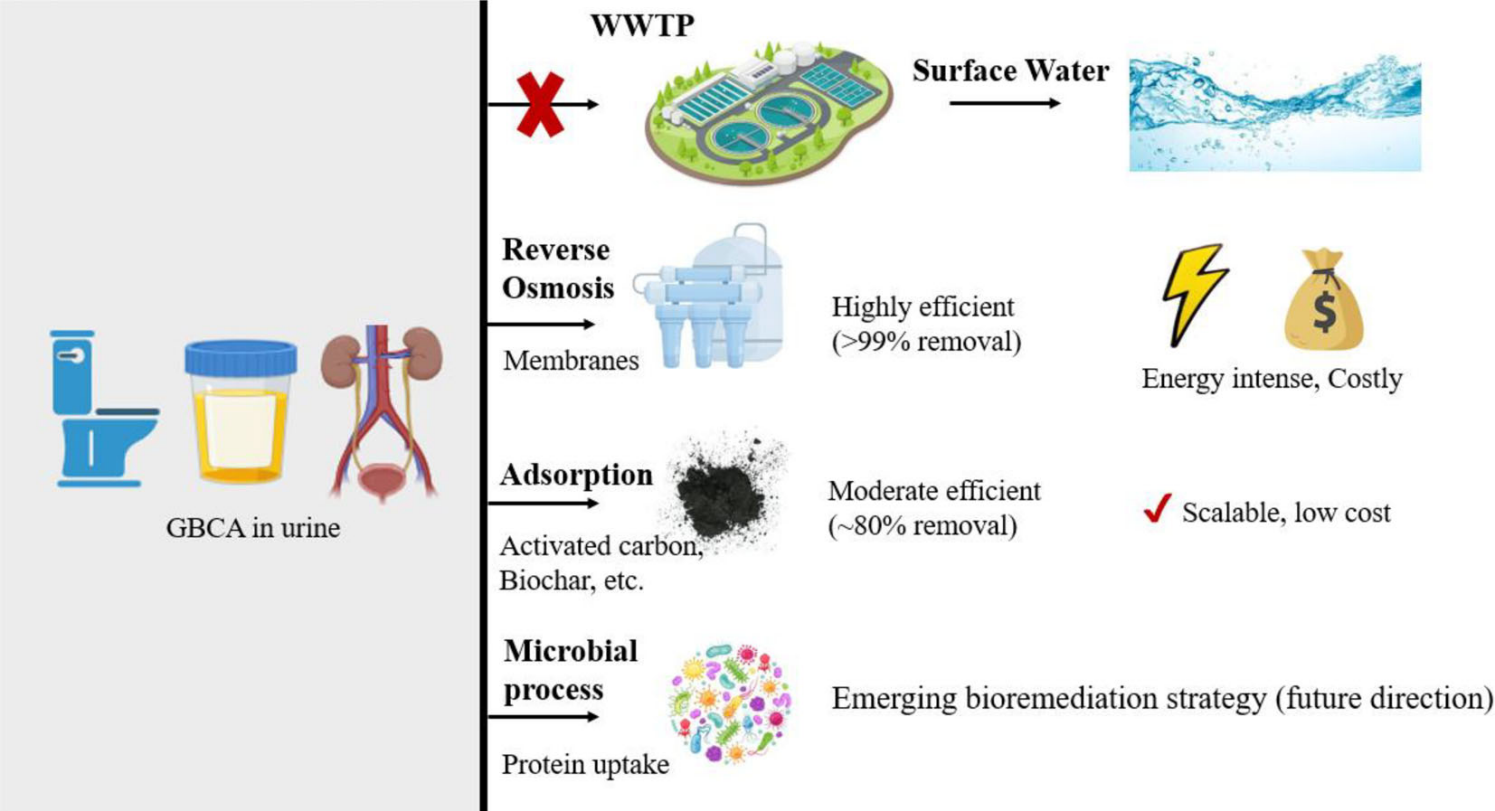
Pathways and treatment strategies for gadolinium‐based contrast agents (GBCAs) excreted in urine and their fate in wastewater treatment plants (WWTPs). Conventional WWTP processes (e.g., activated sludge, sedimentation, and nitrification–denitrification) show negligible GBCA removal due to their high stability and low biodegradability, resulting in persistence through effluent discharge. Advanced physicochemical strategies include reverse osmosis (> 99% rejection, limited by high energy demand and concentrate disposal) and adsorption (˜70%–85% removal depending on sorbent type, with scalability and cost advantages). Emerging biotechnological approaches, such as protein‐mediated uptake and engineered microbial consortia, are being explored as potential pathways for selective and sustainable GBCA sequestration.

Additionally, advanced oxidation processes (AOPs) offer another promising route for degrading certain types of GBCAs, especially linear chelates. Techniques such as UV/peroxide, ozonation, and electrochemical oxidation have demonstrated the ability to degrade GBCAs into more easily removable components, thereby enhancing their overall removal efficiency in wastewater treatment systems [2]. Oturan and Aaron 2014 reported that AOPs leveraging in situ generation of hydroxyl radicals (˙OH) via chemical, photochemical, sonochemical, or electrochemical reaction can significantly degrade GBCAs [77]. Nonetheless, the application of AOPs is often constrained by high energy consumption, operation costs, and the need for specialized infrastructure (Figure 1).

Despite the effectiveness of these advanced technologies, their integration into existing municipal wastewater frameworks remains limited due to concerns about cost, energy use, and long‐term sustainability. Future research should prioritize the development of hybrid treatment systems that combine the strengths of multiple approaches—such as membrane filtration, adsorption, and biological remediation. Integrated strategies offer more practical, scalable, and cost‐efficient solutions for reducing gadolinium contamination in water systems while aligning with broader sustainability goals.

Implementation of these technologies in real‐world settings requires cost and logistical considerations to be incorporated into system design. Reverse osmosis (RO), while capable of removing > 99% of anthropogenic gadolinium [75], is energy‐intensive and has high capital and operational costs, averaging $0.60–$2.50 USD/m^3^ of treated water depending on scale and membrane type [78]. Adsorption‐based approaches using activated carbon, biochar, or ion‐exchange resins offer a low‐cost solution (less than $0.50 USD/m^3^) and require less energy inputs [76], though gadolinium removal efficiencies were lower than RO, around 85% using activated carbon [76]. Both approaches can be retrofitted into existing wastewater treatment plant infrastructure, though performance may vary depending on competing contaminants and the chemical form of gadolinium.

Innovative and emerging approaches need to be developed to collect and treat gadolinium‐rich urine on‐site immediately after MRI procedures. Since up to 95% of administered GBCAs are excreted in urine within 6–24 h post‐injection [38], establishing on‐site urine collection protocols could substantially reduce GBCA loading in municipal wastewater. Pilot studies have demonstrated the technical feasibility of this approach, but challenges related to patient compliance, infrastructure, and regulatory oversight remain. Future research should include techno‐economic assessments of decentralized urine treatment units employing sorbents, nanofiltration, or biological capture modules for on‐site gadolinium capture.

## 4 Discussion

Gadolinium pollution in our bodies and the environment is a complex issue we continue to learn more about with each study. This review summarizes the present evolving state of knowledge and the importance of understanding the causes and repercussions of gadolinium pollution and emphasizes the need for intervention. We also highlight the uncertain health impact of long‐term gadolinium exposure.

### 4.1 Gadolinium‐Chelate Stability Considerations

The acidic dissociation test is an important predictor of in vivo performance, as it indicates a lower propensity to release toxic free gadolinium. A higher kinetic stability of an agent translates to a lower risk of gadolinium retention and accumulation in tissues. The fate of tissue gadolinium remains undetermined. The implications of gadolinium‐chelate stability derive increased relevance given the known persistence. The most vulnerable are children, having the longest potential cumulative exposure time. The risk for gadolinium dissociation is greatest with the linear agents and less with the macrocyclic agents. Even in the hostile in vitro environment of pH 1, the macrocyclic agents have remarkable stability. There is a wide range of stability among the macrocyclic agents. The dissociation half‐life of gadobutrol at a pH of 1 is 18 h, in contrast to gadopiclenol's dissociation half‐life of 20 days under the same conditions [79]. This implies at least 26 times longer stability under the most adverse of conditions. Greater stability could translate to the more complete clearance of these agents from the body with less time for dissociation and long‐term retention. The clinical import of such retention is yet to be determined, so the general strategy is to prevent unnecessary exposure and limit such exposure to when care is impacted.

### 4.2 Reducing Medical Utilization of Gadolinium

There are many unresolved issues warranting further investigation. Chief among these are the efforts to reduce medical utilization of GBCAs through higher efficacy, higher relaxivity agents, and artificial intelligence (AI) and deep learning (DL) MR acquisition techniques permitting lower administered doses per examination. One such AI/DL software product has been FDA and CE approved for MR image enhancement amplification marketed as AiMIFY from Bracco and Subtle Medical, and it is likely other vendors will follow suit [49].

### 4.3 Pollution and Mitigation Strategies

**FIGURE 2:**
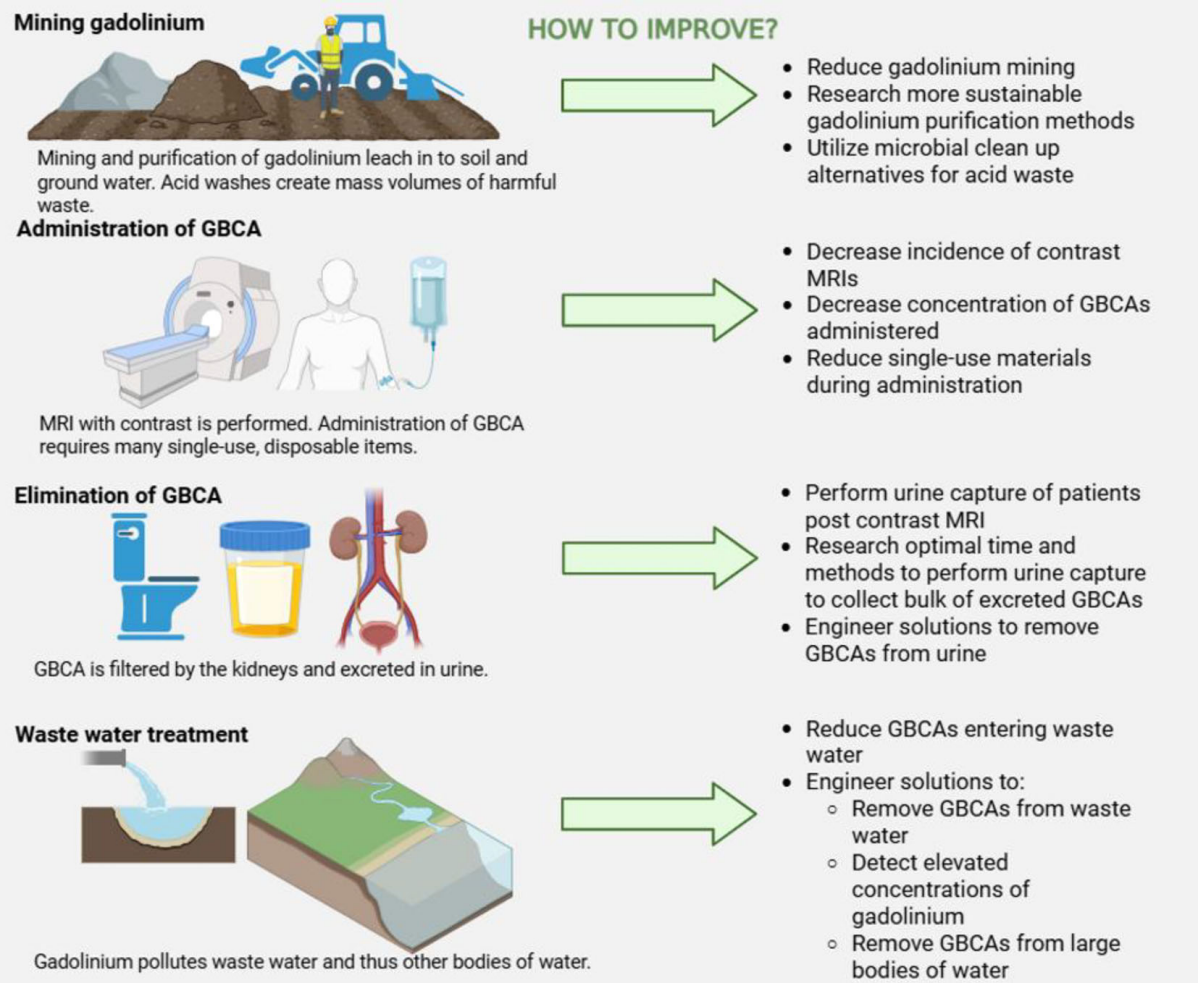
How to reduce gadolinium pollution at each major contaminant source. This figure summarizes the four major points where gadolinium pollution occurs and provides solutions for improvement at each of these levels. GBCA (Gadolinium‐Based Contrast Agent) and MRI (Magnetic Resonance Imaging).

A visual summary of the four main sources of gadolinium pollution is presented in Figure 2 and offers suggestions for mitigating each.

### 4.4 Optimize Mining Gadolinium

We have provided abundant rationale establishing that gadolinium pollution and toxicity is an emerging issue that requires further investigation. The first source of gadolinium pollution comes from the physical mining of gadolinium and the acid washes required to purify it. We recommend gadolinium be mined in a more sustainable manner as suggested in Ognard 2021 and Alonso 2012, although we recognize the limitations of scientists and researchers to control global supply and demand for gadolinium's use in contrast agents and other materials [18, 57]. Additionally, mining for lanthanides can be nonspecific. Thus, complementary methods could be used to further purify acquired mixed mining products. Recently, macrocyclic chelators have been shown to be useful for sustainable aqueous lanthanide separations, that could be applied for separating gadolinium from other lanthanides in mining applications [80].

The most compelling way to mine gadolinium sustainably is to reduce the need for continuous mining. This can be accomplished when other solutions for gadolinium pollution are considered. The concept of circular economy will be pivotal in stopping the compounding issue of gadolinium pollution on human and environmental health. The need for gadolinium mining will decrease if we reduce the incidence of contrast MRIs and the respective concentrations of the contrast, and by capturing and recycling gadolinium for reuse from urine, wastewater, and large bodies of water. By sequestering and recycling gadolinium for further use, less will need to be mined and purified.

### 4.5 Limit Administration of GBCA

Reducing the medical use of GBCAs will have perhaps the broadest impact. The mitigation strategies discussed above including reduced administration, frequency, and dosing of GBCAs for MRIs represent key opportunities to reduce the global burden of gadolinium pollution. The development of higher relaxivity agents such as gadopiclenol and the investigational agent gadoquatrane has equivalent or greater performance at lower administered doses than standard macrocyclic GBCAs [16, 81, 82, 83]. Agents with higher relaxivity will allow a decrease in dose and maintain efficacy [48]. Additionally, deep learning and artificial intelligence (AI) methods are reducing GBCA administration [49]. Recent studies have shown promising results in generating synthetic post‐contrast images from non‐contrast inputs, enabling diagnostic‐quality imaging without conventional gadolinium administration [50, 51, 84, 85, 86]. We applaud the ongoing research using AI and machine learning to reduce the GBCA administered dose to patients while still obtaining high‐quality imaging necessary for diagnoses and appropriate care of patients. Clinically, further reductions in GBCA use could be realized through firm adherence to international guidelines, particularly in market‐oriented healthcare systems where reimbursement structures may inadvertently incentivize overuse. Improved collaboration and education between clinicians and radiologists about appropriate use criteria for contrast in MR imaging will also help reduce excess clinical use. Another opportunity for reduced GBCA use is the use of arterial spin labeling (ASL) which enables perfusion imaging without contrast agents and may increasingly replace dynamic susceptibility contrast techniques in clinical scenarios [87, 88]. The combination of reduced volume and frequency of use, increased agent efficacy, improved image acquisition techniques, and reduction in the amount of residual, unused GBCA in vials and syringes can greatly reduce gadolinium pollution. To date, GBCAs and gadolinium are not classified as toxic waste materials and thus do not require special monitoring or special disposal. Further research must be performed to understand the implications of the way we currently dispose of single‐use materials used during the administration process, expanding on Ognard et al.'s pivotal 2021 study [57].

### 4.6 Elimination of GBCA and Wastewater Treatment

The renal elimination of GBCAs provides opportunities to perform urine capture post‐contrast MRI. Although the duration of renal clearance of GBCAs is related to renal function and hydration status of the patient, we know the majority of GBCAs are excreted with the bulk being in the first hours post‐procedure. By collecting the first several urine samples after contrast administration, we can minimize transfer to typical wastewater facilities which do not currently have the technology to remove gadolinium. There are also opportunities to improve our wastewater treatment to specifically target gadolinium removal and prevent the release of pollution to major bodies of water. We have opportunities to further engineer solutions to remove gadolinium from water once contaminated. Experiments utilizing bacteria as natural gadolinium accumulators are promising developments that could substantially decrease the concentration of this toxic material in our world. The importance of this work, as highlighted by Ebrahimi and Barbieri, is in the potential for gadolinium pollution to be so ubiquitous that it contaminates drinking water and accumulates in meat, seafood, and vegetables [11]. Cost‐benefit analyses of various competing and complementary strategies are required. Prioritization of the development of hybrid treatment systems and the development of integrated strategies driving more practical, scalable, and cost‐efficient solutions for reducing gadolinium contamination are needed, Alignment with broader sustainability goals will flow naturally.

We have also advocated for a particularly innovative targeted collection and treatment of gadolinium‐rich urine following contrast‐enhanced MRI procedures. Since up to 95% of administered GBCAs are excreted in urine within 6–24 h post‐injection [38], establishing on‐site urine collection protocols could substantially reduce GBCA loading in municipal wastewater systems. As doses continue to reduce with advancing technology the immediate capture through these methods may combine to significantly reduce the global burden of pollution. Additionally, we agree with previous mentions of developing an organized monitoring of gadolinium in the environment [11]. What is not measured cannot be managed, a critical metric for understanding the magnitude of anthropogenic gadolinium pollution. The ability to monitor and quantify gadolinium will guide decision making for further research, and allow scientists to identify trends, potential hotspots, and even successes in recycling and management. The review paper Le Fur and Caravan created a summary table that demonstrates the analytical and spectroscopic methods used for investigating and monitoring gadolinium [9]. This table provides a robust summary of each method, the type of sample required, and limitations of each technique. This has value for scientists of all fields as we move forward creating solutions.

### 4.7 Current Developments

Future predictions from the past literature are becoming realized. Lohrke et al. [1, 49] anticipated the development of higher relaxivity contrast agents and improvements in scan acquisition in both speed and sensitivity that are currently state‐of‐the‐art practice. Specifically, the authors are aware of multiple hospital systems in the State of Michigan that either have transitioned to higher relaxivity agents or are planning this switch in large part due to increased efficacy and reduced patient and environmental load. This will result in an immediate 50% reduction in total gadolinium used at these facilities with equivalent diagnostic efficacy [81]. The full potential of high relaxivity agents and the lower limits of dose reduction are yet to be determined. Lohrke et al. also predicted the increased utilization of MR and its expanded role in the assessments of dynamic processes. The clinical deployment of advanced CEST MR techniques is underway at major academic institutions [10]. Translation to community practice is likely as techniques mature and optimal patient management becomes dependent on dissemination of this technology. Dose reduction is the first priority. This can be immediately achieved with the use of the highest relaxivity agents available, coupled with AI and machine learning MR acquisitions, as a bridge to a future state of improved gadolinium ecology and non‐contrast techniques.

## 5 Conclusion

GBCAs have been a pivotal component of MR imaging technology and advancement for diagnoses and guiding treatment. There is compelling interest in reducing the accumulation and potential toxic effects on human and environmental health. The sustainable use of these agents necessitates intervention and innovative engineering to address gadolinium pollution on a global scale. This review compiles the relevant history of gadolinium, improving awareness of the growing evidence of pollution, and the call to action to define necessary steps needed to limit and prevent further pollution. To date, GBCAs and gadolinium are not classified as toxic waste materials even though there is growing evidence to support human and environmental contamination, and no technology exists to remove gadolinium or recycle it. Further research is desperately needed to better understand the long‐term implications of GBCAs, and to push the boundaries of limiting GBCAs without compromising image quality and patient treatment. The future investigation of gadolinium pollution and creating solutions will be a mass collaborative effort across many disciplines such as engineers (chemical, civil, and environmental), radiologists, pharmacists/pharmaceutical companies, and scientists which this review aims to inspire and call to action to minimize the human and environmental risk.

## Conflicts of Interest

Dr. Assaf A. Gilad has a pending patent on technology to capture Gd. LANMODULIN‐BASED PROTEIN Harvey D. Lee, Connor J. Grady and Assaf A. Gilad. US (MSU reference no. is TEC2021‐0056).
